# Association Between Patient Race/Ethnicity, Health Literacy, Socio-Economic Status, and Incidence of Medication Errors: A Systematic Review

**DOI:** 10.1007/s40615-025-02407-8

**Published:** 2025-04-03

**Authors:** Kelly Suen, Sunil Shrestha, Samira Osman, Vibhu Paudyal

**Affiliations:** 1 School of Pharmacy, College of Medical and Dental Sciences, University of Birmingham, Birmingham, B15 2TT UK; 2 Department of Research and Academics, Kathmandu Cancer Center, Tathali, Nala Road, Bhaktapur, Province Bagmati, Nepal; 3 Florence Nightingale Faculty of Nursing, Midwifery and Palliative Care, King’s College, London, UK

**Keywords:** Ethnic minority patients, Health inequality, Health literacy, Medication errors, Socio-economic status

## Abstract

**Introduction:**

Medication errors represent a significant source of healthcare-related harm, leading to mortality, morbidity, and substantial costs to health systems. Existing evidence highlights dissatisfaction and perceived discrimination experienced by patients from minority ethnic and disadvantaged backgrounds within healthcare settings.

**Objective:**

The study aimed to systematically review the literature on associations between patient race/ethnicity, socio-economic status, health literacy, and the incidence or patient experience of medication errors in healthcare and community (home) settings.

**Methods:**

A systematic review was conducted by searching an electronic database including EMBASE, MEDLINE, and PsycINFO. Studies published in English from January 2010 to October 2023, which explored the association between ethnicity, social disadvantage, and medication errors, were included.

**Results:**

Thirteen studies met the inclusion criteria from an initial identification of 2075 titles. Findings indicated that minority ethnic patients were more susceptible to prescription errors, undertreatment, administration errors, and suboptimal medication monitoring and follow-up by healthcare providers. Patients with low health literacy and limited English proficiency were also significantly likely to experience comprehension errors and medication discrepancies. Furthermore, ethnicity and social disadvantages were also associated with an increased risk of overdosing in pediatric medication administration, particularly among parents using dosing cups.

**Conclusion:**

Minority ethnic background, low socio-economic status, and low health literacy can risk a higher likelihood of patients experiencing medication errors. Increasing awareness of these disparities among healthcare staff is essential for developing targeted interventions to mitigate inequalities in patient experiences and care outcomes in relation to medication use and safety. Future research should investigate the importance of intersectionality, such as multiple social disadvantages in this context.

**Supplementary Information:**

The online version contains supplementary material available at 10.1007/s40615-025-02407-8.

## Introduction

The impact of patient race/ethnicity, socio-economic status, and other demographic factors on healthcare quality and outcomes is well-documented, showing that disparities in care are persistent and multifactorial [1]. While healthcare professional guidelines mandate equitable care provision across all patient groups [2–4], evidence demonstrates that implicit negative attitudes among healthcare professionals towards patients of minority ethnic backgrounds are common [5]. Such implicit attitudes can lead to sub-optimal patient-provider communications, as well as biases in diagnostic and treatment choices [6]. Delayed or forgone care are other impacts of perceived racial discrimination [7].

Socio-economic disadvantages such as low income, unemployment, and low formal education are more likely to be experienced by minority ethnic populations, and these can compound barriers to access and outcomes of care [8]. Additionally, language barriers can exacerbate disparities by hindering patient comprehension, leading to medication errors, non-adherence, and lower satisfaction with services [9–11].

Medication errors, which refer to avoidable mistakes in prescribing, transcribing, dispensing, administering, or monitoring medications, pose a serious concern for patient safety, especially in vulnerable groups [12–14]. Medication errors are preventable mistakes that can occur at any point in the medication journey [15]. These errors, which incur an estimated global healthcare expenditure of $42 billion annually, have significant consequences for patients, families, and healthcare systems [16].

Despite medication errors being one of the most common forms of healthcare-related harm, there is a lack of holistic understanding of the importance of race/ethnicity and related inequalities on the prevalence of medication errors. Within previous systematic reviews, medication errors have not been considered within “health outcomes” domain of investigation [6]. In addition to the roles of implicit bias described above, language barriers can exacerbate medication errors [17, 18]. Factors such as language proficiency, beliefs about illness and treatment, and reliance on interpreters increase the risk of patient safety incidents [17]. Additionally, limited health literacy—a patient’s ability to understand and make appropriate health decisions—can increase the risk of medication errors, as patients may misunderstand/misinterpret instructions on proper medication use [19]. Ensuring equitable access to, proper use of, and ongoing monitoring of prescribed medications is essential for reducing healthcare disparities and enhancing patient safety. Understanding how race/ethnicity and social disadvantage are associated with the prevalence and patient experiences of medication errors is key to minimize risk of disproportionate harm and promote equitable care. There have been limited efforts previously to synthesize literature in this area. The systematic review aims to address this gap by examining how race/ethnicity, socio-economic status, language proficiency, and health literacy influence medication errors in both healthcare and community (at home) settings. By synthesizing existing evidence, this review can help raise awareness among healthcare providers and stakeholders while guiding interventions that promote safer and equitable medication use and safety.

## Methodology

Prior to commencing the systematic review, a detailed protocol was developed, and the review was registered on PROSPERO (registration no. CRD42023471330). This review adheres to the PRISMA 2020 guidelines for systematic reviews to ensure thorough and transparent reporting of the methodology and findings [20].

### Inclusion and Exclusion Criteria

Studies published between January 2010 and October 2023 investigating associations between medication errors and race/ethnicity, socio-economic factors, health literacy, and language proficiency were included. All study designs were considered, including observational and cross-sectional designs. Due to resource constraints, only the studies published in English were considered. Studies not addressing medication errors in relation to race/ethnicity, health literacy, language proficiency or socio-economic factors, and non-primary studies (e.g., reviews and opinion pieces) were excluded. Grey literature (e.g., reports, theses, and conference abstracts) was excluded to maintain a focus on high-quality, peer-reviewed studies and ensure methodological rigor.

### Study Identification and Selection

The search was conducted in Medline, EMBASE, and PsycINFO, using combinations of MeSH terms related to medication errors, administration errors, ethnic minorities, and health literacy. The search strategy is presented in Supplementary Material 1**.** Additionally, related systematic reviews were used as references to find relevant keywords in the search strategy [21–25]. Studies were screened by two independent reviewers at the title and abstract levels, followed by full-text screening. Disagreements were resolved by discussion or, if needed, through a third reviewer. The screening process commenced in November 2023.

### Quality Assessment

The emphasis on peer-reviewed articles enabled consistent quality assessment using the Joanna Briggs Institute (JBI) critical appraisal tools, which are well-suited for observational studies﻿—the primary data source in this review [26, 27]. This tool allowed for systematic assessment of bias across various study designs [26, 27].

### Data Extraction and Synthesis

Data on patient race/ethnicity, health literacy, and socio-economic factors (such as income, education, employment)) and their associations with medication errors were extracted. Due to the heterogeneity of study designs and outcomes, a narrative synthesis was conducted.

## Results

### Study Selection andStudy Characteristics

**Fig. 1:**
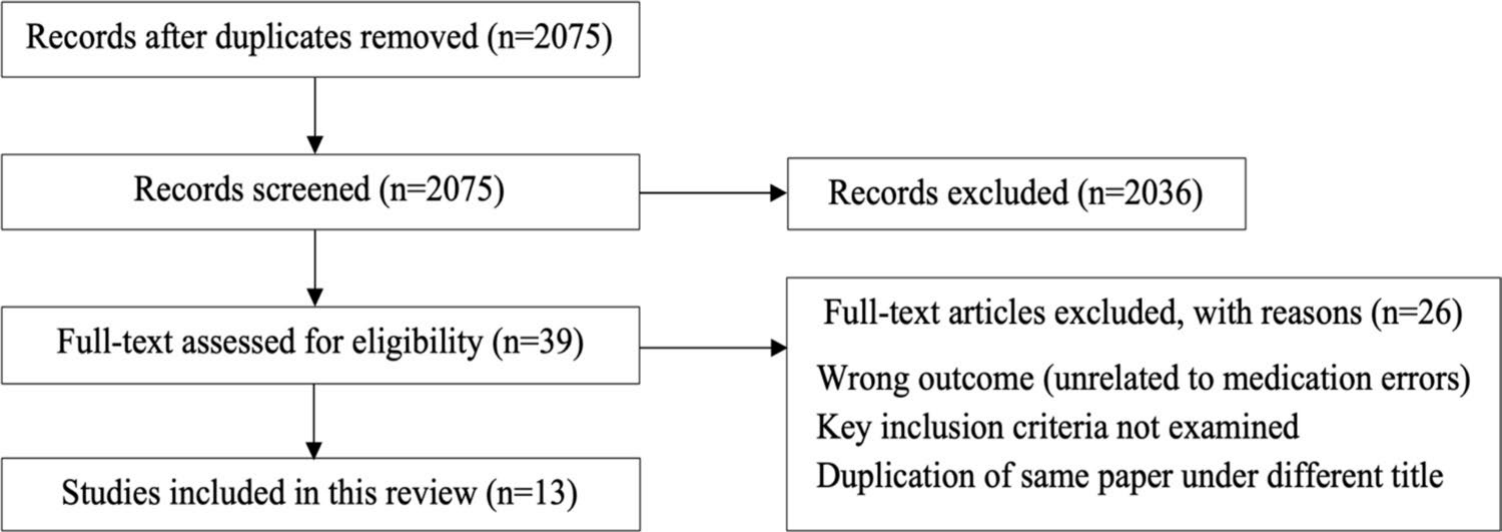
PRISMA flow diagram

From 2075 initial records, 39 studies were screened in full text, resulting in 13 included studies (see Fig. 1). Most studies originated in the USA (*n* = 10) [28–37], with others from Saudi Arabia (*n* = 1) [38], Taiwan (*n* = 1) [39], and the UK (*n* = 1) [40]. The included studies settings included hospital (*n* = 6) [28, 32, 34, 37, 38, 40], patient’s home post-discharge (*n* = 4) [28, 31, 32, 39], general practices (n = 3) [29, 38, 40], home care (*n* = 1) [30], behavioral health clinic (*n* = 1) [33], and pediatric clinics (*n* = 2) [35, 36].

Health literacy level was assessed using the Test of Functional Health Literacy in Adults (short-form) or the Newest Vital Sign (NVS). Studies focused on parents’/carers’ health literacy and administration errors, and the NVS was used due to its validity in both English and Spanish [34–36]. Additional socio-economic data, including ethnicity, education, and income, were collected through self-reports.

### Characteristics of Participants Within Included Studies

The profiles of the participants are presented in Table 1, and these varied across the included studies. For instance, Mixon et al. (2014) [28] reported a mean participant age of 59, with a predominantly White (81%) and African American (17%) individuals, while Glick et al. (2019) [34] included primarily Hispanic (74%) and Black (12%) parents of children under 12, with 76% reporting low health literacy. Education levels also varied, with certain groups displaying high proportion of participants of limited formal education—such as the Taiwanese cohort in Liang et al. (2022) [39], where 64.4% lacked formal education or completed only elementary schooling. Other studies highlighted the prevalence of socio-economic challenges, including uninsured status in the study by Stewart and Lynch (2012) [29] and high levels of homelessness in the study by Marks et al. (2012) [33].

**Table 1:** Characteristics of study participants

Study	Participants	Age (mean)	Ethnicity	Gender	Socio-economic characteristics of study participants
Mixon et al., 2014 [28]	471	59 (± 12.5)	African American (17%), White (81%), all other races (2%)	Male: 52.1%, Female: 47.9%	Mean social support score: 25.7 (± 4.3). 9.8% had 17 + years of education; 34.4% had 13–15 years. Income: 18.3% earned $25,000–34,999, 7.2% below $10,000, and 8.3% over $100,000
Stewart & Lynch, 2012 [29]	219	47.4 (± 11.9)	White (57%), Black (36%), Latino (3%), Other/Unknown (4%)	Female: 62%, Male: 38%	Entirely indigent and uninsured population
Hu et al., 2012 [30]	82	81.18 (± 7.5)	Chinese Americans (100%)	Female: 55%, Male: 45%	79.3% had less than a high school education. 23% lived alone post-discharge; 38 reported English proficiency
Akbarov et al., 2015 [40]	205,519	38.89 (± 7.96)	Not specified	Female: 49%, Male: 51%	Mean IMD: 37.03 (± 10.19)
Roth et al., 2011 [31]	200	60 +	White (50%), Black (50%)	Female: 77%, Male: 23%	Most participants from White ethnicity groups attended college or postgrad. 64 White vs. 49 Black participants lived alone.
Liang et al., 2022 [39]	1988	60 +	Taiwanese (100%)	Female: 51%, Male: 49%	64.4% had no formal education or only elementary. High prevalence of mobility aids: 373 were bedridden, 320 with devices, and 279 use wheelchairs.
Lindquist et al., 2011 [32]	254	79.3 (± 6.4)	White (69%), African American (26%), Asian (2%), Hispanic & Other (3%)	Female: 53%, Male: 47%	Over half had a college education; 32.3% had inadequate health literacy.
Marks et al., 2012 [33]	426	44.7 (± 10.2)	White, Black, Other	Female: 53%, Male: 47%	95% uninsured; 24.2% homeless. Others lived in transitional housing or with friends/family.
Almazrou et al., 2014 [38]	575	Mothers of children ≤ 12 years	Saudi Arabian (100%)	Female: 100%	29% had secondary education, and 36% had college/postgrad. Varied education levels, from illiterate to college graduates
Glick et al., 2019 [34]	165	Parents of children ≤ 12 years	Hispanic (74%), Black (12%), Asian (7%), White & Other (7%)	Female: 93%, Male: 7%	87% of children had public insurance; 76% of parents had low health literacy. Most earned under $25,000
Harris et al., 2017 [35]	2110	Parents of children ≤ 8 years	Hispanic (54%), non-Hispanic (46%)	Not specified	69.2% had limited English proficiency, and 82.7% had limited health literacy. 43.4% had education below high school
Williams et al., 2019 [36]	493	Parent: 30.4 (± 7.7), Child: 2.3 (± 2.4)	Hispanic (41%), White (6%), Black (33%), Other (14%)	Children: Female 48%, Male 52%	48.5% of families had an income below $20,000. 66.3% had limited health literacy; 27.1% did not complete high school.
Rungvivatjarus et al., 2023 [37]	29	Parents of children ≤ 12 years	African (7%), Asian (3%), Caucasian (21%), Latino (55%), Mixed/Other (14%)	Female: 86%, Male: 14%	The primary language is Spanish (41%) or English (41%). Some parents had limited English proficiency.

IMD: Index of Multiple Deprivation

### Study Quality

Results of the quality assessment are summarized in Supplemental Materials 2 and 3. All cross-sectional studies (*n* = 8) included detailed participant and setting descriptions and appropriate statistical analysis, while the cohort studies documented follow-up times and reasons for attrition. However, many studies lacked sufficient adjustment for confounding factors, potentially impacting outcome reliability.

### Race/Ethnicity and Medication Errors

Eight studies examined racial and ethnic disparities in medication errors (Table 2). Seven of these studies showed that patients from minority ethnic/racial backgrounds were at a disadvantage compared to the White majority. Common themes emerged, including increased non-adherence, undertreatment, and suboptimal medication monitoring among ethnic minority patients. For instance, the study by Roth et al. (2011), which included in-home reviews and medication records evaluation of community-dwelling older adults, reported that Black patients experienced more medication-related problems than White patients, with a mean of 6.23 problems compared to 4.9 (*P* < 0.001). This was despite White patients used more medications on average than Black patients (11.6 vs. 9.7; *P* < 0.001) [31]. In the study by Mixon et al. (2014) [28], African-American participants showed higher odds of post-discharge medication discrepancies than White participants (OR 2.02; 95% CI 1.13–3.60). Similarly, Stewart and Lynch (2012) [29] observed that a higher proportion of patients of Black ethnicity (79%) experienced at least one medication discrepancy compared to White patients (71.8%) in the outpatient setting. However, the difference was not statistically significant (Table 2).

**Table 2:** Summary of main findings in included studies

Author, year	Country	Participants	Type	Setting	Duration	Main findings
Mixon et al., 2014 [28]	Nashville, USA	471	Prospective cohort	Hospital & post-discharge	Oct 2011 to Aug 2012 (11 months)	Higher health literacy (OR 0.84; 95% CI 0.74–0.95) was linked with lower odds of misunderstanding medication instructions Non-white/Black participants (OR 2.02; 95% CI 1.13–3.60) showed higher odds of medication discrepancies Increased educational attainment (OR 1.29 per 4-year increment; 95% CI 1.06–1.56) correlated with higher odds of medication errors due to commission.
Stewart & Lynch, 2012 [29]	Pittsburgh, USA	219	Observational case series	General practice	2009–2010 (13 months)	Among patients from White ethnicity groups, 71.8% had at least one medication discrepancy (MD) compared to 79% of patients from Black ethnicity (*P* = 0.092). Patients remembering over 75% of their medication details had fewer MDs (*P* = 0.015) No significant correlation was observed between MDs and knowledge of medication name, indication, or regimen (*P* = 0.246, 0.214, 0.553, respectively)
Hu et al., 2012 [30]	New York City, USA	82	Cross-sectional	Home care	June 2010 to July 2011 (13 months)	No correlations were found among English and Chinese proficiency, sex, marital status, and living status with the occurrence and number of potentially inappropriate medications and medication discrepancies
Akbarov et al., 2015 [40]	Salford, UK	205,519	Cross-sectional	General practice/hospital	Jan–June 2012 (6 months)	In deprived areas (IMD ≤ 28.9), patients had higher odds of missed monitoring than in less deprived areas (IMD > 46.1; aOR 3.1; 95% CI 1.74–5.53) Those with IMD ≥ 50 saw a 9.01% prevalence of missed monitoring.
Roth et al., 2011 [31]	North Carolina, USA	200	Prospective cohort	Participant homes	12 months	Patients of White ethnicity used more medications (mean: 11.6 vs 9.7; *P* < 0.01), but patients of Black ethnicity experienced more medication-related problems (mean: 6.2 vs 4.9; *P* < 0.01)
Liang et al., 2022 [39]	Taipei, Taiwan	1988	Cohort	Participant homes	Apr 2016 to Mar 2019 (35 months)	Age, living alone, and socio-economic disadvantages contributed to insufficient disease and treatment knowledge (*n* = 291), self-care skills (*n* = 336), and lifestyle issues (*n* = 248)
Lindquist et al., 2011 [32]	Chicago, USA	254	Survey	Hospital/post-discharge	June 2007 to Feb 2009 (20 months)	Patients with low health literacy faced higher non-adherence rates (47.7%) than those with marginal (31.8%) or adequate literacy (20.5%; *P* = 0.002). Participant race data collected but unclear whether association between race and non-adherence was evaluated
Marks et al., 2012 [33]	Richmond, USA	426	Retrospective cohort	Behavioral clinic	Oct 2008 to Sep 2009 (11 months)	Patients from Black ethnic groups experienced more medication-related problems (213) than White (130; *P* = 0.0006), including ineffective therapy and adverse effects
Almazrou et al., 2014 [38]	Riyadh, Saudi Arabia	575	Cross-sectional	Hospital/clinic	Mar–Apr 2013 (1 month)	Mothers with low health literacy overdosed more using dosing cups than those with high school or college education (*P* < 0.001). Mothers with higher educational attainment underdosed less frequently.
Glick et al., 2019 [34]	New York City, USA	165	Prospective cohort	Hospital	June 2015 to Apr 2017 (22 months)	Low health literacy was associated with comprehension (aOR 1.8; *P* = 0.002) and adherence errors (aOR 1.5; *P* = 0.03), though not significant after adjusting for comprehension (aOR 1.2; *P* = 0.4). Participant race data collected but unclear whether association between race and medication errors.
Harris et al., 2017 [35]	New York, USA	2110	Cross-sectional	Pediatric clinic	Aug 2013 to Dec 2014 (16 months)	Spanish-speaking parents had higher dosing error odds (aOR 1.6; CI 1.3–1.9) than English speakers. Those with limited health literacy/English proficiency were twice as likely to err in dosing.
Williams et al., 2019[36]	New York, USA	493	Cross-sectional study	Pediatric outpatient clinic	20th Feb 2015 to 23rd July 2015 (5 months)	Parents with limited health literacy were more likely to use dosing cups when administering medications (aOR 2.4; 95% CI 1.2–4.6) instead of oral syringes. Older parent age, being non-US born, race/ethnicity, Spanish language, and site were not associated with the belief that dosing cups are best for dosing accuracy.
Rungvivatjarus et al., 2023 [37]	California, USA San Diego, USA	45	Qualitative study	Hospital	Dec 2019 to Sept 2020 (9 months)	Poor communication between caregivers and providers regarding prescribed medications including drug name, dosage, frequency, and purpose was common in an ethnically diverse and deprived community. Some parents lacked knowledge regarding the role of a child’s medications including medication name and dosage, expected side effects, and dosing instructions.

OR: Odds Ratio, aOR: Adjusted Odds Ratio, CI: Confidence Interval.

### Socio-Economic Status and Medication Errors

Socio-economic status emerged as an important predictor of medication errors, particularly in settings serving underserved or deprived populations. One study [40] found that primary care practices in high-deprivation areas were three times more likely to miss essential monitoring steps, such as INR tests for patients on warfarin, compared to practices in low-deprivation areas (OR 3.1; 95% CI 1.74–5.53) [40]. Another study conducted in Taiwan showed that patients with lower socio-economic status were significantly more likely to store medications inappropriately and administer them incorrectly (*P* < 0.001) [39]. This study significantly improved medication adherence, storage practices, and self-care knowledge (*P* < 0.001) following pharmacists’ interventions, highlighting the effectiveness of pharmacist-led home visits’ role in mitigating medication errors in disadvantaged populations.

### Health Literacy and Medication Errors

A consistent finding across the included studies was the correlation between low health literacy and increased medication errors. For instance, patients with limited health literacy had higher post-discharge medication discrepancies, with 51% of patients having at least one medication and 59% reporting misunderstandings in a study of patients hospitalized for acute coronary symptoms [28]. Higher health literacy reduced these risks (OR = 0.84, CI 0.74-0.95). A qualitative study reported that comprehension issues due to low health literacy were associated with higher non-adherence and dosing errors in parental populations, particularly among parents with limited knowledge of medication names and dosages [37].

Higher parental education levels were associated with reduced pediatric medication administration errors [34–36]. The study by Almazrou et al. (2014) [38] showed that overdosing errors with dosing cups were more prevalent among mothers with limited or no education compared to those with high school or college degrees (*P* < 0.001). The study by Glick et al. (2019) demonstrated that parents with lower health literacy, often associated with lower education levels, were 8.7 times more likely to make adherence errors due to poor comprehension of medication instructions (aOR = 8.7; 95% CI, 5.9–12.9) [34]. Williams et al. (2019) reported that parents with limited health literacy were 2.4 times more likely to use dosing cups, a tool associated with higher error rates, compared to parents with higher literacy (aOR = 2.4 [1.2–4.6]) [36].

### Impact of Limited English Proficiency on Medication Errors

Limited English proficiency was a risk factor for paediatric medication administration errors as reported in the included studies. In the study by Harris et al., Spanish-speaking parents were 1.6 times more likely to make a dosing error compared to English-speaking parents (adjusted odds ratio [aOR] 1.6, 95% CI 1.3–1.9) [35]. Conversely, in Hu et al. (2012) [30], no association was found between English or Chinese proficiency and the prevalence of medication discrepancies or potentially inappropriate medications (PIMs) among older Chinese Americans. Other factors, such as polypharmacy, were more predictive of medication discrepancies in this cohort.

## Discussion

This study explored the roles of race/ethnicity, socio-economic factors, health literacy, and English proficiency on the prevalence of medication errors. Our findings highlight that patients from ethnic minority groups faced a disproportionate risk of medication errors, particularly in relation to prevalence and experience of medication discrepancies (e.g., incorrect medication documented in patient records) and medication-related problems (e.g., inappropriate drugs prescribed). The review also highlights the importance of socio-economic disadvantage in the prevalence and patient experience of medication errors, as patients living in deprived areas had greater chances of missing medication monitoring than those living in affluent areas. In addition, socio-economic disadvantages also contributed to insufficient knowledge regarding prescribed treatments. The role of health literacy was mainly studied in the context of parent administration of medication to their children. Low health literacy and lower educational attainment were associated with pediatric dosing errors and non-adherence.

Ethnic minority patients experience distinct challenges that increase their risk of medication errors. In addition to the role of implicit bias described in the literature [5], language barriers and cultural differences often hinder effective communication between healthcare providers and patients, impacting comprehension of medication instructions [41]. Misunderstandings, mainly when instructions are not delivered in the patient’s preferred language or lack cultural relevance, can result in non-adherence, dosing mistakes, and misinterpreting medical guidance [41, 42].

Socio-economic factors, such as income, employment, and education, are critical determinants of access to healthcare resources and medication safety [43, 44]. Financial limitations often reduce patients’ ability to afford continuous care, follow-up visits, and medication adherence aids. For instance, one of the included studies showed that primary care practices in high-deprivation areas are more likely to miss essential medication monitoring compared to those in low-deprivation areas (OR = 3.1, 95% CI 1.74–5.53)[40]. Our findings indicate that low health literacy is associated with an elevated risk of medication errors, affecting various aspects of medication management, including dosing, adherence, and regimen complexity. Specifically, low health literacy contributes to parental difficulties in managing pediatric medications, leading to misunderstandings of instructions (e.g., dosing frequency), confusion about medication names, and non-adherence due to misconceptions about treatment necessity [37]. Patients with limited literacy will likely struggle to comprehend complex medication schedules, recognize side effects, and properly use medication tools like dosing cups and syringes [37, 38]. Moreover, limited digital literacy further restricts access to reliable health information or digital resources [45]. Therefore, healthcare professionals must adopt tailored strategies, such as using pictorial instructions and simplified dosing tools, to enhance medication safety for parents with low literacy levels.

### Implications for Practice and Policy

Enhancing awareness among healthcare providers regarding the association between race, ethnicity, and socio-economic factors on medication safety is key to promoting patient safety. Disparities in outcomes are likely linked to implicit bias amongst service providers, in addition to the roles of structural inequalities. Future studies should investigate this phenomenon in the context of medication errors. Incorporating cultural competence and health disparity training into healthcare education and ongoing professional development can help practitioners recognize these risks and proactively address them in clinical practice.

Improving patient-provider communication is key to reducing medication errors, especially among parents with low health literacy to enhance pediatric medication safety. Evidence-based communication strategies, such as the “teach-back” method, where patients repeat back instructions to confirm understanding, have been shown to improve comprehension and adherence [46]. Implementing structured communication tools and pictorial dosing instructions could help bridge gaps in understanding, reduce medication errors, and improve patient safety. Expanding access to interpretation services and culturally tailored patient education materials is also essential to enhance medication safety for vulnerable groups and bridge communication gaps.

### Strengths

A key strength of this systematic review is its comprehensive examination of the association between patient race/ethnicity, socio-economic factors, health literacy, and the prevalence of medication errors. Validated tools were used for quality assessment, which helped ensure a rigorous quality evaluation of included studies. Additionally, the adherence to the PRISMA guidelines [20] throughout this systematic review enhances the transparency and reproducibility of the methods and findings.

### Limitations

Most studies were conducted in the USA, which may limit the generalizability of findings to other regions with differing healthcare systems and cultural dynamics. The relatively small sample sizes of several studies included in this review may also restrict the robustness of some findings. Additionally, the reliance on cross-sectional designs in many studies prevents causal inferences regarding the relationship between socio-economic factors and medication errors, highlighting a need for more longitudinal research. Furthermore, the primary data collection method for medication discrepancies was patient self-reporting, which may introduce social desirability and recall biases. Another limitation is the underrepresentation of fathers in studies on pediatric administration errors, which may limit insights into the broader familial context and generalizability across all caregivers. Further research is needed to address these limitations—such as by using larger, more diverse populations, incorporating longitudinal designs, and including varied caregiver perspectives.

## Conclusion

This study provides an understanding that patients from ethnic minorities/races, lower socio-economic backgrounds and health literacy are disproportionately impacted by medication errors. Wider literature links these disparities to the negative implicit attitude of providers in the context of minority race/ethnicity. Continuous cultural competency training, integrated into educational curricula and professional development activities should focus on the impact of patient characteristics and socio-economic disadvantages on medication safety. Tailored resources for patients with low literacy, low socio-economic status, and those facing language barriers can mitigate inequalities. Additionally, expanding access to follow-up support through community pharmacies is critical for providing reliable post-discharge guidance, particularly in underserved areas.

## Supplementary Information

Below is the link to the electronic supplementary material.

ESM 1 (DOCX 42.9 KB)

## Data Availability

All data relevant to the study have been reported in the manuscript and supplementary materials.
