# Supplementary material for: Association Between Patient Race/Ethnicity, Health Literacy, Socio-Economic Status, and Incidence of Medication Errors: A Systematic Review
Source: J Racial Ethn Health Disparities. 2025 Apr 3;13(3):2161–72. doi: 10.1007/s40615-025-02407-8 (PMC13157403; doi:10.1007/s40615-025-02407-8)
Supplement: Supplementary file 1 — (DOCX 42.9 KB) [file 40615_2025_2407_MOESM1_ESM.docx]

# Supplementary material 1: Full Search Strategies Used in the Literature Searching

Database: Ovid MEDLINE

1. Medication Errors/ or medication error*.mp. (15971)
2. Prescribing error*.mp. (770)
3. Dispensing error*.mp. (287)
4. Administration error*.mp. (808)
5. ((medication* or transcrib* or prescrib* or dispens* or administ*) adj3 (incident* or mistake* or error*)).mp. [mp=title, book title, abstract, original title, name of substance word, subject heading word, floating sub-heading word, keyword

heading word, organism supplementary concept word, protocol supplementary concept word, rare disease supplementary concept word, unique identifier, synonyms, population supplementary concept word, anatomy supplementary concept word] (17735)

1. medication incident*.mp. (152)
2. Healthcare Disparities/ or Health Equity/ or Health Status Disparities/ or Culturally Competent Care/ or Social Determinants of Health/ or Sociology, Medical/ (51205)
3. (disadvantaged or discriminat* or disparat* or disparit* or disproportion* or inequal* or inequit* or unequal or underserved or under-served or (cultural* adj3 compet*) or (social* adj3 determin*)).ti,ab,kf. (483687)
4. (South Asian* or ethnic* or race* or racial* or minority or minorities or "people of color" or African-American* or Black* or Hispanic* or Chican* or Latino* or Latina* or Latinx or Mexican-American* or Asian-American* or Chinese-

American or Filipino* or Japanese or Korean or Vietnamese or Native American* or Indian* or indigenous).ti,ab,kf. (783712)

1. (healthcare disparit* or impoverish* or homeless* or immigrant* or inequality* or social determinant or indigent or

low-income or low-wage or lower-income or socioeconomic difference* or social class or Medicaid or Medicare or migrant* or poverty or social position* or social hierarchy or educational level* or educational attainment* or (public adj (assistance or housing)) or social or socio* or SES or undocumented or uninsured or veteran*).mp. or (working adj2 (class or poor)).ti,ab,kf. [mp=title, book title, abstract, original title, name of substance word, subject heading word, floating sub-heading word, keyword heading word, organism supplementary concept word, protocol supplementary concept word, rare disease supplementary concept word, unique identifier, synonyms, population supplementary concept word, anatomy supplementary concept word] (1386963)

1. health literacy.mp. or Health Literacy/ or Educational Status/ (73734)
2. Ethnic Groups/ or Minority Groups/ or African Americans/ or Arabs/ or Asian Americans/ or Hispanic Americans/ or Mexican Americans/ or Indigenous Peoples/ (178767)
3. Minority Groups/ or Asian People/ or Black People/ or BAME.mp. (129047)
4. Socioeconomic Factors/ or Economic Status/ or Employment/ or Homeless Persons/ or Medicaid/ or Medically

Uninsured/ or exp Medicare/ or Poverty/ or Poverty Areas/ or Public Assistance/ or Public Housing/ or Social Class/ or "Transients and Migrants"/ or Undocumented Immigrants/ or Veterans/ or Working Poor/ (406629)

1. Asylum seekers.mp. or Refugees/ (13656)
2. 1 or 2 or 3 or 4 or 5 or 6 (17735)
3. 7 or 8 or 9 or 10 or 11 or 12 or 13 or 14 or 15 (2475801)
4. 16 and 17 (1559)
5. limit 18 to (english language and yr="2010 -Current") (975)

Database: Embase, APA PsycInfo

1. Medication Errors/ or medication error*.mp. (24915)
2. Prescribing error*.mp. (2606)
3. Dispensing error*.mp. (747)
4. Administration error*.mp. (1683)
5. ((medication* or transcrib* or prescrib* or dispens* or administ*) adj3 (incident* or mistake* or error*)).mp. [mp=ti, ab, hw, tn, ot, dm, mf, dv, kf, fx, dq, tc, id, tm] (31067)
6. medication incident*.mp. (400)
7. Healthcare Disparities/ or Health Equity/ or Health Status Disparities/ or Culturally Competent Care/ or Social Determinants of Health/ or Sociology, Medical/ (88749)
8. (disadvantaged or discriminat* or disparat* or disparit* or disproportion* or inequal* or inequit* or unequal or underserved or under-served or (cultural* adj3 compet*) or (social* adj3 determin*)).ti,ab,kf. (959721)
9. (South Asian* or ethnic* or race* or racial* or minority or minorities or "people of color" or African-American* or Black* or Hispanic* or Chican* or Latino* or Latina* or Latinx or Mexican-American* or Asian-American* or Chinese-

American or Filipino* or Japanese or Korean or Vietnamese or Native American* or Indian* or indigenous).ti,ab,kf.

(1794494)

1. (healthcare disparit* or impoverish* or homeless* or immigrant* or inequality* or social determinant or indigent or low-income or low-wage or lower-income or socioeconomic difference* or social class or Medicaid or Medicare or

migrant* or poverty or social position* or social hierarchy or educational level* or educational attainment* or (public adj (assistance or housing)) or social or socio* or SES or undocumented or uninsured or veteran*).mp. or (working adj2 (class or poor)).ti,ab,kf. [mp=ti, ab, hw, tn, ot, dm, mf, dv, kf, fx, dq, tc, id, tm] (3376115)

1. health literacy.mp. or Health Literacy/ or Educational Status/ (143038)
2. Ethnic Groups/ or Minority Groups/ or African Americans/ or Arabs/ or Asian Americans/ or Hispanic Americans/ or Mexican Americans/ or Indigenous Peoples/ (359626)
3. Minority Groups/ or Asian People/ or Black People/ or BAME.mp. (161918)
4. Socioeconomic Factors/ or Economic Status/ or Employment/ or Homeless Persons/ or Medicaid/ or Medically

Uninsured/ or exp Medicare/ or Poverty/ or Poverty Areas/ or Public Assistance/ or Public Housing/ or Social Class/ or "Transients and Migrants"/ or Undocumented Immigrants/ or Veterans/ or Working Poor/ (579056)

1. Asylum seekers.mp. or Refugees/ (27283)
2. 1 or 2 or 3 or 4 or 5 or 6 (31067)
3. 7 or 8 or 9 or 10 or 11 or 12 or 13 or 14 or 15 (5559500)
4. 16 and 17 (3256)
5. limit 18 to (english language and yr="2010 -Current") (2449)
6. remove duplicates from 19 (2423)
7. limit 20 to "remove medline records" (1100)

**Supplementary material 2:Checklist for analytical cross-sectional studies (n=8)**

| **Study** | Were the criteria for inclusion in the sample clearly defined? | Were the study subjects and the setting described in detail? | Was the exposure measured in a valid and reliable way? | Were objective, standard criteria used for measurement of  the condition? | Were confounding factors identified? | Were strategies to deal with confounding factors stated? | Were the outcomes measured in a valid and  reliable way? | Was appropriate statistical analysis used? | Total Yes Scores |
| --- | --- | --- | --- | --- | --- | --- | --- | --- | --- |
| **Cross-sectional studies** | | | | | | | | | |
| **Stewart & Lynch, 2012** | Yes | Yes | Yes | Yes | No | No | Yes | Yes | 6 |
| **Hu et al., 2012** | Yes | Yes | No | Yes | No | No | Yes | Yes | 4 |
| **Akbarov et al., 2015** | Yes | Yes | Yes | Yes | No | No | Yes | Yes | 5 |
| **Lindquist et al., 2011** | Yes | Yes | Yes | Yes | No | No | Yes | Yes | 6 |
| **Almazrou et al., 2014** | Yes | Yes | Yes | No | No | No | No | Yes | 4 |
| **Harris et al., 2017** | Yes | Yes | Yes | Yes | No | No | Yes | Yes | 5 |
| **Williams et al., 2019** | Yes | Yes | Yes | Yes | No | No | Yes | Yes | 5 |
| **Rungvivatjarus et al., 2023** | Yes | Yes | No | No | Yes | No | No | Yes | 4 |

**Supplementary material 3: Joanna Briggs Institute critical appraisal checklist for analytical cohort studies (n=5)**

| **Study** | Were the two groups similar and recruited from the same population? | Were the exposures measured similarly to assign people to both exposed and unexposed  groups? | Was the exposure measured in a valid and reliable way? | Were confounding factors identified? | Were strategies to deal with confounding factors stated? | Were the groups/participants free of the outcome at the start of the study (or at the moment of exposure)? | Were the outcomes measured in a valid and reliable way? | Was the follow up time reported and sufficient to be long enough for outcomes to occur? | Was follow up complete, and if not, were the reasons to loss to follow up described and  explored? | Were strategies to address incomplete follow up utilised? | Was appropriate statistical analysis used? | Total Yes Scores |
| --- | --- | --- | --- | --- | --- | --- | --- | --- | --- | --- | --- | --- |
| **Cohort studies** | | | | | | | | | | | | |
| **Mixon et al., 2014** | Not applicable | Not applicable | Yes | No | No | Not applicable | Yes | Yes | Yes | No | Yes | 5 |
| **Roth et al., 2011** | Not applicable | Not applicable | Yes | No | No | Not applicable | Yes | Yes | Yes | No | Yes | 5 |
| **Liang et al., 2022** | Not applicable | Not applicable | Yes | No | No | Not applicable | No | Yes | Yes | No | Yes | 4 |
| **Marks et al., 2012** | Not applicable | Not applicable | No | No | No | Not applicable | Yes | Yes | Yes | No | Yes | 4 |
| **Glick et al., 2019** | Not applicable | Not applicable | Yes | No | No | Not applicable | Yes | Yes | Yes | No | Yes | 5 |
